# Synthetic adiponectin-receptor agonist, AdipoRon, induces glycolytic dependence in pancreatic cancer cells

**DOI:** 10.1038/s41419-022-04572-8

**Published:** 2022-02-04

**Authors:** Sharon J. Manley, Appolinaire A. Olou, Jarrid L. Jack, Mariana T. Ruckert, R. McKinnon Walsh, Austin E. Eades, Bailey A. Bye, Joe Ambrose, Fanuel Messaggio, Shrikant Anant, Michael N. VanSaun

**Affiliations:** 1 Department of Cancer Biology, University of Kansas Medical Center, Kansas City, KS United States; 2 Department of Surgery, University of Miami Miller School of Medicine, Sylvester Comprehensive Cancer Center, Miami, FL United States

**Keywords:** Pancreatic cancer, Cell growth, Biologics, Pancreatic cancer

## Abstract

Obesity creates a localized inflammatory reaction in the adipose, altering secretion of adipocyte-derived factors that contribute to pathologies including cancer. We have previously shown that adiponectin inhibits pancreatic cancer by antagonizing leptin-induced STAT3 activation. Yet, the effects of adiponectin on pancreatic cancer cell metabolism have not been addressed. In these studies, we have uncovered a novel metabolic function for the synthetic adiponectin-receptor agonist, AdipoRon. Treatment of PDAC cells with AdipoRon led to mitochondrial uncoupling and loss of ATP production. Concomitantly, AdipoRon-treated cells increased glucose uptake and utilization. This metabolic switch further correlated with AMPK mediated inhibition of the prolipogenic factor acetyl coenzyme A carboxylase 1 (ACC1), which is known to initiate fatty acid catabolism. Yet, measurements of fatty acid oxidation failed to detect any alteration in response to AdipoRon treatment, suggesting a deficiency for compensation. Additional disruption of glycolytic dependence, using either a glycolysis inhibitor or low-glucose conditions, demonstrated an impairment of growth and survival of all pancreatic cancer cell lines tested. Collectively, these studies provide evidence that pancreatic cancer cells utilize metabolic plasticity to upregulate glycolysis in order to adapt to suppression of oxidative phosphorylation in the presence of AdipoRon.

## Introduction

Pancreatic cancer is the fourth leading cause of cancer death with a dismal five year survival rate of 9% [1]. Around 90% of pancreatic cancer is the more lethal pancreatic ductal adenocarcinoma (PDAC) [2]. The current verified risk factors for pancreatic cancer include smoking, alcohol consumption, aging, pancreatitis, and obesity [3, 4]. Of these, the rising rate of obesity is positively correlated with the increasing incidence of pancreatic cancer [5–8]. While obesity harbors dysregulation of multiple physiological systems, we have demonstrated that dysregulation of adipose-derived cytokines, termed adipokines [9], directly influences pancreatic cancer proliferation, migration, and tumor growth [10–12]. Other studies have demonstrated that adipokines have additional pleiotropic roles in cancer progression, including acting as inflammation mediators, growth factors, and angiogenic factors [9, 11, 13, 14].

Adiponectin is an adipokine secreted at high levels from adipose tissue of the lean population [15–18]. Cellular signaling in response to binding of adiponectin to its receptors, AdipoR1 and AdipoR2 [19–22], is mediated by APPL1 (adaptor protein-containing pleckstrin homology domain, phosphotyrosine-binding domain, and leukine zipper motif), an adaptor protein known to link adiponectin receptors to downstream adiponectin-signaling pathways [23]. Key downstream adiponectin mediators include the energy-sensing protein AMP kinase (AMPK) and the peroxisome proliferator-activated receptor (PPAR) [24]. As an inhibitor of anabolic processes and an activator of catabolic processes such as lipid breakdown, adiponectin can suppress proliferation and induce an anticancer response. Accordingly, clinically low plasma adiponectin levels are associated with increased cancer risk [25–27] and adiponectin is considered to be antitumorigenic from studies that showed it reduced tumorigenic activity in breast [28–31], endometrial [32–34], colorectal [35–37], prostate [38–41], and pancreatic cancers [25]. The mechanism by which adiponectin exerts its antitumorigenic effects is not clear, but while adiponectin-mediated AMPK signaling has been implicated in certain cancers [41], alternate mechanisms through JAK-STAT suppression have been shown [42, 43]. Additionally, adiponectin may also directly affect the mitogen-activated protein-kinase (MAPK) pathway via activation of the stress-induced p38 MAPK or suppression of pERK [43].

AdipoRon is a synthetic analog that mimics the actions of adiponectin by stimulating both AdipoR1 and AdipoR2 [22] to subsequently activate an increase in p-AMPK and PPAR similar to endogenous adiponectin [22]. Animal studies using AdipoRon found that, just like adiponectin, it ameliorates insulin resistance, diabetes, and inflammation with a maximal circulating value of 11.8 µM [22]. Furthermore, AdipoRon also has antiproliferative and anticancer properties [44]. With regard to pancreatic cancer, we demonstrated that the effects of adiponectin and AdipoRon on cancer cells were partially due to suppression of the STAT3 signaling pathway as well as AMPK-pathway activation resulting in decreased proliferation [10, 11]. Interestingly, other studies showed that AMPK activation in response to AdipoRon correlated with a suppression of insulin resistance and glucose intolerance [22], highlighting a metabolic therapeutic potential for AdipoRon.

Alterations in metabolism are widely defined as a hallmark of cancer, and this holds true for PDAC [45]. The highly heterogeneous tumor microenvironment shapes PDAC cellular metabolic rewiring [46, 47]. Additionally, high desmoplasia causes unique metabolic challenges for PDAC, such as hypovascularity, hypoxia, and nutrient deprivation, that result in metabolic reprogramming [48]. Cancer cells typically adapt to low-energy conditions through the Warburg effect, where cancer cells preferentially utilize glycolysis over mitochondrial oxidative phosphorylation to compensate for energy deficiencies [49, 50]. However, some cancer cells contradict Warburg’s postulation and exhibit fully intact and functioning mitochondria and are metabolically dynamic [48, 51]. For example, after inactivation of KRAS, a subpopulation of PDAC tumor cells relied on mitochondrial respiration and beta-oxidation to survive, which eventually led to tumor relapse [52]. While the evidence points to intrinsic metabolic plasticity in pancreatic cancer cells, their metabolic adaptations in response to adipose-derived factors have not been fully investigated.

In this paper, we demonstrate that PDAC cells readily switch from mitochondrial respiration to glycolysis in response to stimulation with AdipoRon. This switch is associated with the AMPK phosphorylation-mediated inhibition of acetyl coenzyme A carboxylase 1 (ACC1), yet AdipoRon treatment fails to alter lipid catabolism. The results support a modulatory role for AdipoRon on the metabolic phenotype of pancreatic cancer and further demonstrate that dual metabolic targeting of glycolysis in conjugation with AdipoRon may provide an effective antiproliferative strategy.

## Results

### AdipoRon attenuates mitochondrial respiration in PDAC cell lines

**Fig. 1:**
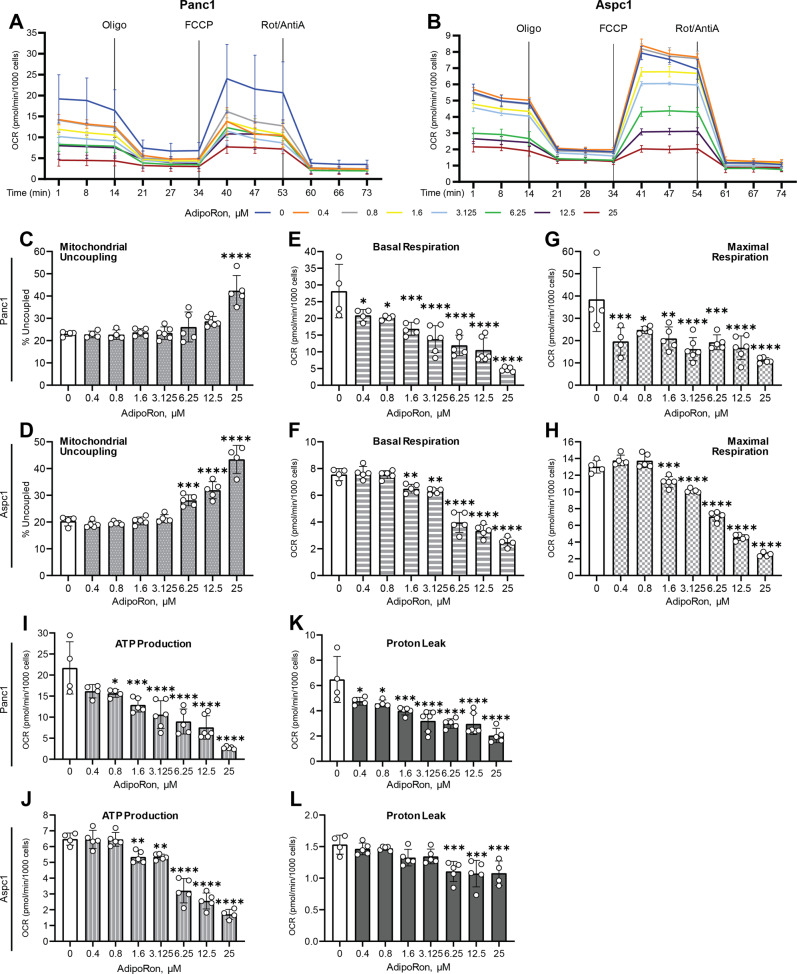
AdipoRon suppresses metabolic parameters in a dose-dependent manner. Seahorse XFe96 bioanalyzer was used to measure oxygen-consumption rate (OCR) in Panc1 (**A**) and Aspc1 cell lines (**B**) either untreated (0) or pretreated with 0.4, 0.8, 1.6, 3.125, 6.25, 12.5, or 25 μM AdipoRon for 45 min, with initial basal measurements followed by sequential injections of oligomycin, carbonyl cyanide-4-(trifluoromethoxy)phenylhydrazone (FCCP), and rotenone/antimycin A. Mitochondrial uncoupling (**C**, **D**), basal respiration (**E**, **F**), maximal respiration, (**G**, **H**), ATP production (**I**, **J**), and proton leak (**K**, **L**) were calculated from the OCR measurements in (**A**, **B** respectively). One-way ANOVA, *n* ≥ 3; error bars, SD; *P < 0.05.

**Fig. 2:**
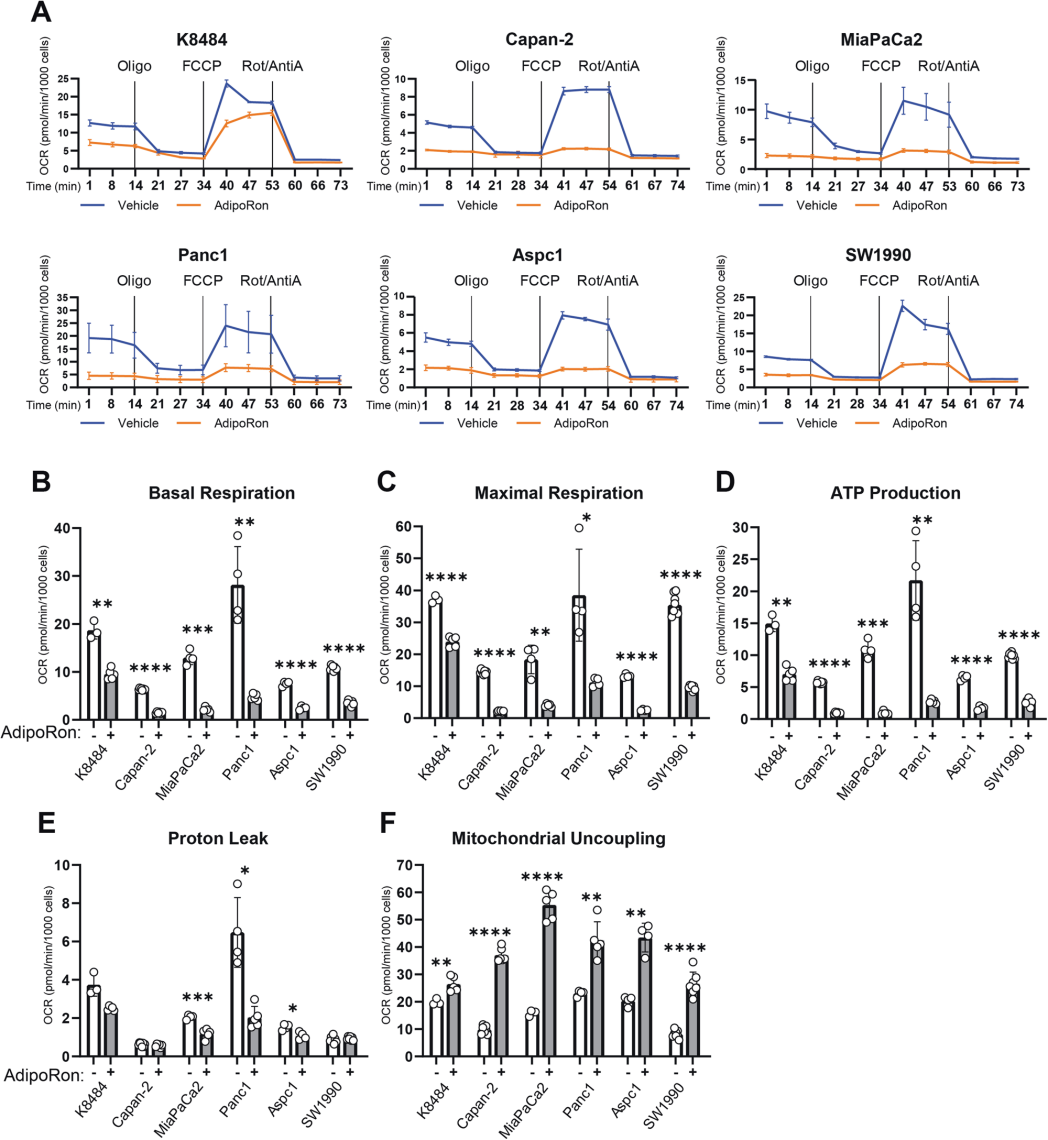
AdipoRon inhibits mitochondrial respiration and ATP production in pancreatic cancer cells. Seahorse XFe96 bioanalyzer was used to measure oxygen-consumption rate (OCR) in six different PDAC lines (**A**) either untreated or pretreated with 25 μM AdipoRon for 45 min, with initial basal measurements followed by sequential injections of oligomycin, carbonyl cyanide-4-(trifluoromethoxy)phenylhydrazone (FCCP), and antimycin A and rotenone. Basal respiration (**B**), maximal respiration (**C**), ATP production (**D**), proton leak (**E**), and mitochondrial uncoupling (**F**) were calculated from the OCR measurements in (**A**) for the respective cell lines. Statistics: *t*-test, *n* ≥ 3; error bars, SD; *P < 0.05.

Our initial observations revealed a visual acidification of culture media from AdipoRon-treated pancreatic cancer cell cultures (Supplementary Figure 1), suggesting a potential increased glycolytic activity. To assess the impact of AdipoRon on mitochondrial function, we exposed multiple PDAC cell lines to AdipoRon and assessed alterations in basal metabolic parameters using a Seahorse-based assay. We first performed AdipoRon dose–response studies to evaluate alterations in mitochondrial respiration. Two PDAC cell lines (Panc1 and Aspc1) were initially exposed to serially diluted AdipoRon-containing media ranging from 0.4 to 25 μM for 45 min prior to the initiation of the assay, to prime the cells. The assay results demonstrated that AdipoRon treatment significantly decreased the oxygen-consumption rate (OCR) in both cell lines (Fig. 1A, B) in a dose-dependent manner. Measurements for multiple mitochondrial respiration parameters were then derived from the OCR, which includes mitochondrial uncoupling, basal respiration, maximal respiration, proton leak, and ATP production. Mitochondrial uncoupling occurs when the electron-transport chain is not a primary driver of ATP synthase activity. AdipoRon treatment increased mitochondrial uncoupling (Fig. 1C, D) in PDAC cell lines, suggesting that mitochondrial integrity is compromised. Additionally, AdipoRon treatment not only significantly decreased mitochondrial basal and maximal respiration (Fig. 1 E–H), resulting in greatly diminished ATP production (Fig. 1 I and J), but also attenuated proton leak in the cells (Fig. 1K and L). Decreased proton leak is another indicator of diminished mitochondrial activity. We expanded our analysis to include multiple PDAC cell lines (see supplementary table for information about the cell lines) using a single dose of AdipoRon (25 µM). In these additional PDAC cells too, we found that AdipoRon treatment decreased the OCR (Fig. 2A), basal and maximal respiration (Fig. 2B, C), ATP production (Fig. 2D) and proton leak (Fig. 2E) while increasing mitochondrial uncoupling (Fig. 2F) in all PDAC cell lines tested, as compared with no treatment control (Fig. 2). Exceptionally, the K8484 cells were slightly resistant to AdipoRon-mediated decreases in mitochondrial respiration (Fig. 2A) when compared against the other PDAC cell lines. While reduction of proton leak was evident in the MiaPaCa2, Panc1, and Aspc1 cell lines in response to AdipoRon, this was not exhibited by the K8484, Capan-2, and SW1990 cells (Fig. 2E). Taken together, the results indicated that AdipoRon treatment led to an attenuation of mitochondrial respiration.

### AdipoRon treatment upregulates glycolysis in PDAC cell lines

**Fig. 3:**
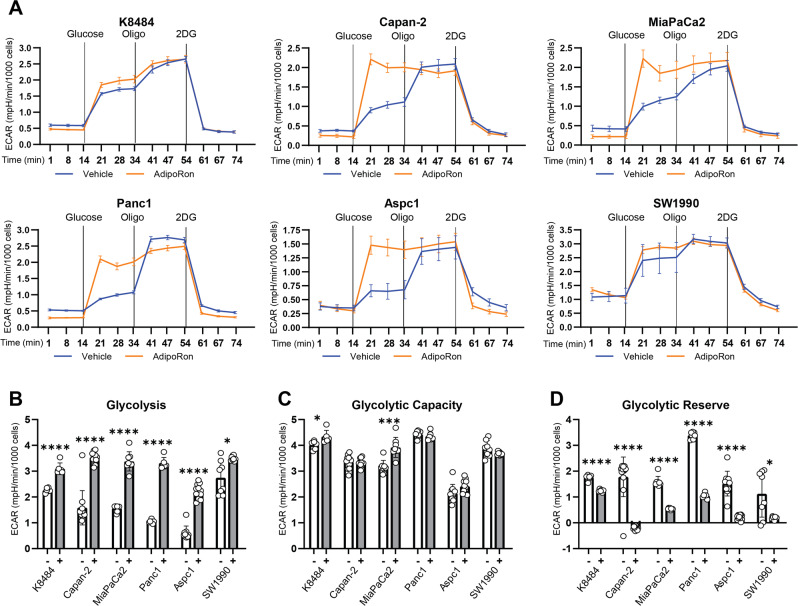
AdipoRon activates glycolysis in pancreatic cancer cell lines. Seahorse XFe96 bioanalyzer was used to measure extracellular acidification rate (ECAR) in six different PDAC lines (**A**) either untreated (vehicle) or pretreated with 25 μM AdipoRon for 45 min, with initial basal measurements followed by sequential injections of glucose (10 mM), oligomycin (2 µM), and 2-deoxy-d-glucose (2-DG) (50 mM). Glycolysis (**B**), glycolytic capacity (**C**), and glycolytic reserve (**D**) were calculated from the ECAR measurements in (**A**) for the respective cell lines. Statistics: *t*-test, *n* ≥ 3; error bars, SD; *P < 0.05.

As described in the figures above, AdipoRon treatment decreased OCR and mitochondrial ATP production. Anaerobic glycolysis, not involving mitochondrial respiration, is a less-efficient ATP-producing bioenergetic pathway that is frequently activated to compensate for deficient mitochondrial ATP synthesis that is the more efficient ATP-generating process in cells [50, 53]. We further performed a Seahorse-based glycolysis-stress test to assess glycolytic parameters in six PDAC cell lines and to determine whether anaerobic glycolysis was being upregulated to compensate for deficiencies in mitochondrial respiration and ATP production. In brief, extracellular pH was measured sequentially to monitor the extracellular acidification rates, ECAR. The values were then used to calculate the rate of glycolysis, glycolytic capacity, and glycolytic reserve. AdipoRon treatment altered ECAR response in all six PDAC cells tested (Fig. 3A), correlating with increased glycolysis (Fig. 3B), suggesting a compensatory response to the defective mitochondrial ATP production. AdipoRon increased glycolytic capacity in only two of the six cell lines tested, while no significant change in glycolytic capacity was noted in the other four cell lines (Fig. 3C). Furthermore, AdipoRon treatment depleted glycolytic reserves in pancreatic cancer cells (Fig. 3D), indicating that because of impaired mitochondria, the cells relied on utilization of the glycolytic process. These results further demonstrate that AdipoRon treatment leads to activation and maximization of the glycolytic process in pancreatic cancer cell lines.

### AdipoRon treatment increases glucose uptake

**Fig. 4:**
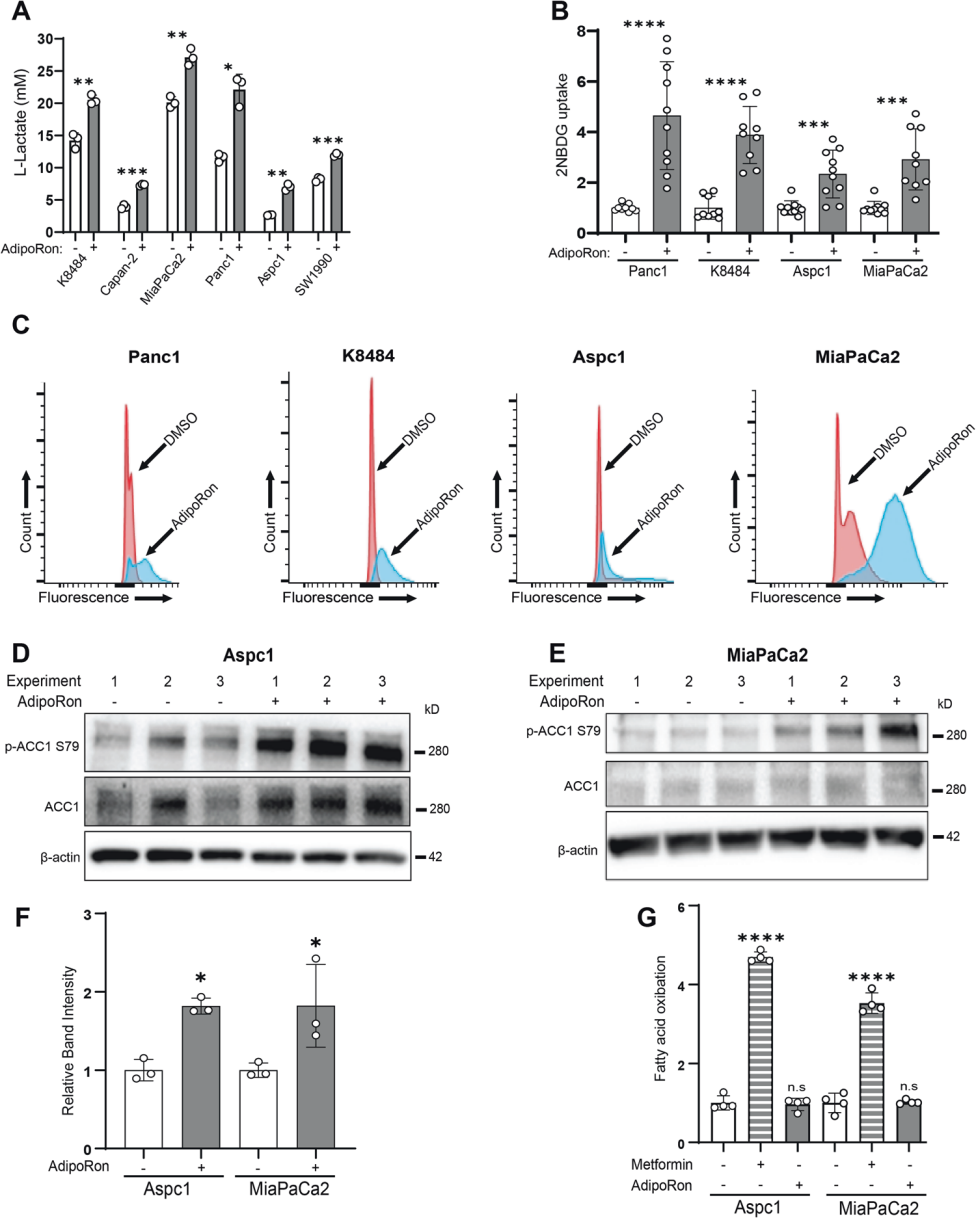
AdipoRon increases glucose utilization. **A** PDAC cells were cultured in reduced serum (2.5% fetal bovine serum) conditions for 24 h prior to treatment with 25 μM AdipoRon for 48 h and assessed for levels of secreted lactate (*n* = 3). **B**, **C** PDAC cells were treated with AdipoRon for 48 h, and then glucose- and serum-starved for 40 min prior to incubation with 2NBDG (200 µM) for 80 min. At the endpoints, the cells were washed and assessed for 2NBDG content either by plate reading (excitation 475 nm, emission 550 nm) or flow-cytometric analysis (**B**, *n* = 10; **C**, *n* = 4). **D–F** Levels of phospho-ACC1 were assessed in response to AdipoRon treatment (6 h), blots were cropped, and levels adjusted for clarity (*n* = 3). **G** Fatty acid oxidation was measured following AdipoRon treatment (6 h), *n* = 4. Statistics: *t*-test (**F**) or one-way ANOVA (**G**); error bars, SD; *P < 0.05.

To further characterize the results above, we postulated that AdipoRon treatment will shift PDAC cellular bioenergetic demand toward a dependency on anaerobic glycolysis. We first assessed glycolysis-related proteins Glut1, Glut4, hexokinase I and II, PKM I and II (as well as their phosphorylated forms), PFKP, and LDHA in AdipoRon-treated pancreatic cancer cell lysates by western blot. While the levels of Glut1 and Glut4 appeared to not change in any of the cell lines (Supplementary Fig. 2), K8484 and Aspc1 cell lines showed a slight increase in hexokinase 1 and LDHA, respectively; no significant changes were detected in any other glycolytic proteins across all four cell lines (Supplementary Fig. 3A). Consistent with the acidification of the media (Supplementary Fig. 1) and the augmented glycolysis (Fig. 3) induced by AdipoRon treatment, all six cell lines showed statistically significant increases in lactate secretion into the conditioned media after treatment with AdipoRon (Fig. 4A). These results suggested that the AdipoRon-mediated increase in glycolysis may not be due to an increase in the levels of the enzymatic proteins involved but rather to an augmented glucose entry into the cells. To confirm altered glucose uptake in the AdipoRon-treated cells, we measured internalization of fluorescent-labeled glucose, 2NBDG. Total fluorescence analysis showed that the AdipoRon-treated cells increased uptake of 2NBDG (Fig. 4B), which was further corroborated by flow-cytometric analysis (Fig. 4C and Supplementary Fig. 3B).

AdipoRon has pleiotropic effects on cell metabolism, including its activation of the energy-sensing AMPK pathway [24]. Previous studies have shown that AMPK activity can also suppress aerobic glycolysis [54] and induce fatty acid oxidation via phosphorylation-mediated inhibition of acetyl coenzyme A carboxylase 1 (ACC1) [55]. Western blot analysis revealed that AdipoRon treatment of PDAC cell lines increased the levels of p-AMPK and p-ACC1, although the increase in p-AMPK was not consistent across all cell lines assessed (Supplementary Figure 3C). Therefore, we functionally validated the increase in p-ACC1 levels in the Aspc1 and MiaPaCa2 cell lines (Fig. 4D–F). Fatty acid beta oxidation (FAO) was measured in response to AdipoRon as well as Metformin, the widely known activator of AMPK-mediated FAO. While Metformin treatment confirmed induction of FAO in both PDAC cell lines, no change was observed in AdipoRon-treated cells (Fig. 4G). Together, our data suggest that AdipoRon treatment results in enhanced glycolysis without inducing lipolysis.

### Glucose deprivation enhances the growth-suppressive effects of AdipoRon

**Fig. 5:**
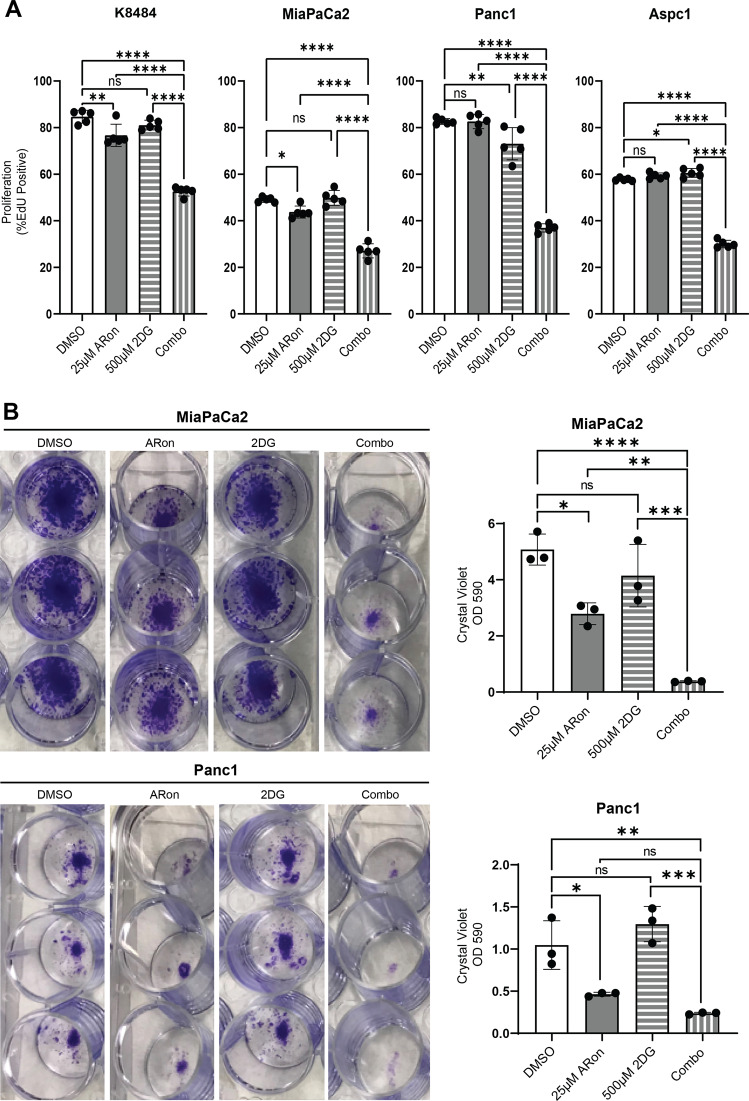
Disruption of glycolysis enhances AdipoRon-mediated growth inhibition. Indicated cells were treated alone or in combination with AdipoRon (25 µM) and/or the glycolysis inhibitor 2DG for 24 h and then proliferation was measured by EdU incorporation (**A**, *n* = 5). MiaPaCa2 and Panc1 cells were pulse-treated with AdipoRon alone or in combination with 2DG for 36 h, allowed to recover, and assessed for colony formation 7 days later by crystal violet staining (**B**, *n* = 3). One-way ANOVA; error bars, SD; *P < 0.05.

Considering that AdipoRon treatment impaired mitochondrial function and caused the cells to maximize their glycolytic capacity, we wanted to determine whether persistence of pancreatic cancer cells in the presence of AdipoRon was dependent on anaerobic glycolysis. For this, we exposed AdipoRon-treated PDAC cells to the glycolysis inhibitor, 2-deoxy-D-glucose (2DG). Cell growth was measured by EdU incorporation or by colony-forming ability of the cells. When compared with control, the AdipoRon alone showed partial growth suppression while the 2DG alone showed no change, which was evident by a decreased EdU incorporation and colony formation (Fig. 5A, B). The combination of AdipoRon and 2DG produced an additional statistically significant decrease in growth for both of the assays when compared with their control. To determine whether this phenomenon is applicable to other cancer types, we exposed the murine breast cancer cell model, 4T1, to AdipoRon or 2DG or a combination of both, which synergistically suppressed the growth of the breast cancer cell line 4T1 (Supplementary Fig. 4A). Based on these results, we posited that AdipoRon would be more effective at impairing cancer cells when the cells are in glucose-deprived conditions. To test that, we treated the cells with AdipoRon in low-glucose (10 mM) or glucose-free media. Assessment of cell survival demonstrated that AdipoRon combined with low glucose significantly decreased cell survival in all the PDAC cell lines (Supplementary Fig. 4B), including the 4T1 murine breast cancer cell line (Supplementary Fig. 4C). The results support a role for glucose utilization as a compensatory survival program when the cells are exposed to AdipoRon.

## Discussion

Obesity is well known to create metabolic disturbances through increased energy intake and utilization, but obesity can also lead to reactivity in the white adipose. A major consequence of reactive adipose is an alteration in the production of adipokines [10–12]. While many of these adipokines promote cancer development, some of them suppress the cellular hallmarks of cancer. Our previous study reported that adiponectin as well as its receptor mimetic, AdipoRon, can effectively inhibit proliferation and induce apoptosis in pancreatic cancer cell lines, albeit at concentrations greater than 25 µM [10]. We also demonstrated that the effects of adiponectin and AdipoRon on cancer cells were due, in part, to suppression of the STAT3 signaling pathway as well as AMPK-pathway activation [10, 12]. Importantly, the energy-sensing AMPK pathway has emerged as a critical arm of metabolic rewiring in cancer cells, suggesting that, by activating the AMPK pathway, adiponectin signaling may affect cancer cell metabolism.

In order to develop a deeper understanding of the mechanism of action for adiponectin in pancreatic cancer progression, we investigated the effect of its synthetic agonist, AdipoRon, on PDAC cellular metabolism. We demonstrated that sublethal AdipoRon treatment induced mitochondrial uncoupling (Figs. 1C, D and 2F) and decreased proton leak (Figs. 1K, and 2E), indicating significant mitochondrial dysfunction. As a result, all mitochondrial parameters tested, oxygen consumption, basal and maximal respiration, and ATP generation, were negatively impacted (Fig. 1A, B; 1E–J; Fig. 2A–D). Concomitantly, AdipoRon-treated cells increased their glucose uptake (Fig. 4B, C; Supplementary Fig. 3B), in correlation with an increased extracellular cellular acidification (Fig. 3A), lactate secretion (Fig. 4A), and glycolysis (Fig. 3B). Glut1 and 4 receptor levels did not change (Supplementary Fig. 2), hinting that potential alternate mechanisms or Glut-receptor isoforms may be mediating the increased glucose uptake into the pancreatic cancer cell lines after AdipoRon treatment. Additionally, we did not detect any significant changes in the level of multiple proteins involved in the glycolytic process, except for hexokinase 1 and LDHA, which were slightly increased in two of our cell lines (Supplementary Fig. 3A). This suggested that the enhanced glycolysis in response to AdipoRon was not the result of increased protein levels but rather an augmented glucose entry into the cells and/or enzymatic activity, yet, the mechanism for this remains to be determined. Together, these results suggest that during impaired mitochondrial function, pancreatic cancer cells default to anaerobic glycolysis, which they relied on and maximized for survival, as their glycolytic reserve was decreased during AdipoRon exposure (Fig. 3D). Disruption of glycolysis in AdipoRon-treated cells decreased their ability to form colonies and correlated with decreased proliferation (Fig. 5) and survival (Supplementary Fig. 4). Our data indicated that survival of PDAC cells (Supplementary Fig. 4B), and potentially other cancer cell types (Supplementary Fig. 4A; C), in the presence of AdipoRon, depends on their metabolic flexibility to switch from the more energetically efficient glycolysis, to an incomplete and fermentative glycolysis.

Previous studies indicated that AMPK activity can suppress aerobic glycolysis [54] and induce lipolysis [55]. The AdipoRon-induced metabolic switch we observed in pancreatic cancer cells was associated with an increased AMPK and ACC1 phosphorylation, although p-AMPK was not consistent across all the cell lines (Supplementary Fig. 3C; Fig. 4D–F). ACC1 is a prolipogenic protein whose phosphorylation is known to inhibit lipid synthesis and induce fatty acid oxidation [56–58]. Although we detected increased pACC levels in response to AdipoRon, our FAO assay did not reveal an induction of lipolysis (Fig. 4G). This could be explained by the fact that when AdipoRon induces mitochondrial dysfunction in the cancer cells, FAO is a lesser option for energy generation by the cancer cells, since such energy generation would require functional mitochondria. Additional experiments would be needed to conclusively delineate the necessity for AMPK and ACC1 activity in this context. However, it is possible that AMPK–ACC1 is enhancing the anti-oxidant capacity of AdipoRon-treated cells [55]. Studies have reported that AdipoRon treatment led to accumulation of superoxide in pancreatic cancer [43], a precursor of oxidative stress. Likewise, our unpublished results showed increased ROS levels in AdipoRon-treated cells. These stress-derived molecules have potentially deleterious effects in cells that would cause the cells to deploy their anti-ROS-defense mechanism. In light of the literature, we speculate that part of that mechanism could involve anti-oxidants generated through the AMPK–ACC1 axis such as NADPH [55]. The latter is primarily generated from the pentose-phosphate pathway (PPP) and mitochondrial metabolism. But given that AdipoRon disrupted mitochondrial function, there is a possibility that cells may have activated an AMPK–ACC1-dependent but a mitochondria-independent mechanism for redox balance. To this point, glycolytic intermediates such as glucose-6-phosphate can be used as substrates in the PPP.

Last, the data presented here align with published results demonstrating inhibition of cancer by AdipoRon but are the first to provide a mechanistic association implicating a glycolytic switch in pancreatic cancer. Understanding how AdipoRon inhibits PDAC cells’ mitochondrial function and energy metabolism, we can now speculate that the low expression of adiponectin in cancer [25–27] as well as the low level of adiponectin-receptor expression in pancreatic cancer [10] could be driven by a metabolic survival adaptation. Suppression of adiponectin signaling in pancreatic cancer may represent a mechanism to keep mitochondrial stress low and mitochondrial activity optimal. Identifying and targeting the players in this metabolic adaptation that repress the adiponectin ligand or its receptor expression could be key to addressing obesity and obesity-driven pancreatic cancer. Future studies would also explore the therapeutic potential for combining AdipoRon with a glycolysis inhibitor for pancreatic cancer.

## Materials and methods

### Cell culture and reagents

Capan-2, MiaPaCa2, Panc1, Aspc1, and SW1990 were purchased from ATCC (Manassas, VA, USA). Capan-2 is a human primary tumor-derived cell line containing a KRAS^G12V^ mutation. MiaPaCa2 and Panc1 are primary human tumor-derived PDAC cell lines expressing mutant KRAS, mutant p53, and deletion of CDK. Aspc1 (mutant KRAS, mutant p53, and deletion of CDK) and SW1990 (mutant KRAS, wild-type p53, and deletion of CDK) are metastatic human PDAC cell lines. K8484 (KRas^G12D/+^; p53^R172H/+^) murine cell line has been previously described [59]; a table in the supplementary materials provides a summary of this information. Capan-2, Aspc1, and SW1990 were cultured in high-glucose (4.5 g/L) RPMI-1640 medium (ThermoFisher Scientific, Waltham, MA USA, cat# A1049101) supplemented with 10% heat-inactivated fetal bovine serum (FBS, Atlanta Biologicals/RD Systems, Atlanta, GA, USA) and antibiotic–antimycotic (Thermo Fisher Scientific, cat# 15240062). MiaPaCa2, Panc1, and K8484 were cultured in high-glucose (4.5 g/L) Dulbecco’s Modified Eagle Media (ThermoFisher Scientific cat# 11-995-073) supplemented with 10% heat-inactivated FBS (R&D Systems–Atlanta Biologicals) and antibiotic–antimycotic (ThermoFisher Scientific). K8484 cells isolated from the KPC mouse were acquired from Dr. Tuveson and validated by PCR as well as the presence of KrasG12D by western blot. K8484 cells were maintained in high-glucose DMEM, 5% FBS, and antibiotic–antimycotic. 4T1 murine mammary cells were acquired from ATCC and maintained in DMEM, 5% FBS, and antibiotic–antimycotics. AdipoRon was purchased from Cayman Chemical (cat# 15941). Oligomycin, carbonyl cyanide 4-(trifluoromethoxy) phenylhydrazone (FCCP), rotenone, antimycin A, and D-Glucose were purchased from Sigma-Aldrich (St. Louis, MO USA); 2-deoxyglucose was from ThermoFisher.

### Mitochondrial and glycolysis stress tests utilizing Seahorse XFe96 Bioanalyzer

K8484, MiaPaCa2, Panc1, Aspc1, and SW1990 were seeded at a density of 4 × 10^4^ cells per well and Capan-2 was seeded at a density of 1 × 10^4^ cells per well in Seahorse XF96 cell culture microplates and incubated for 24 h at 37 °C/5% CO_2_. Mito-stress test and glycolysis stress test were performed according to the manufacturer’s protocols (Agilent/Seahorse Biosciences, Santa Clara, CA, USA). Briefly, the cells were washed two times with medium and then treated with sequential concentrations of AdipoRon (0, 0.4, 0.8, 1.6, 3.125, 6.25, 12.5, and 25 µM) for 45 min prior to the assay. For cell quantification, bright-field scanning of cells was performed with the Cytation1 (BioTek) during the non-CO2 incubation period. After the assay, cells were stained with Hoechst 33342 (injected along with oligomycin during the assay) and scanned with Cytation1. The measurements were then normalized per 1000 cells counted.

### Mito stress test

Oxygen-consumption rate (OCR) was measured (pmol/min) at 12 time points with sequential injections of either oligomycin, an ATP-synthase inhibitor (1 µM for K8484 and MiaPaCa2 and 2 µM for rest of the PDAC cells), or carbonyl cyanide 4-(trifluoromethoxy) phenylhydrazone (FCCP, a mitochondrial uncoupling agent, 1 µM), or rotenone (complex-I inhibitor, 50 µM), or antimycin A (complex-III inhibitor, 50 µM). The mitochondrial parameters were calculated according to the manufacturer’s instructions. Negative values for these parameters were excluded from their group.

### Glycolysis stress test

Extracellular acidification rate (ECAR) was measured (mpH/min) at 9 timepoints with sequential injections of 10 mM glucose, 1 or 2 µM oligomycin, and 50 mM 2-deoxyglucose. The calculations for glycolytic parameters followed the manufacturer’s instructions. Negative values were excluded from their group.

### Western Blot and antibodies

Cells were lysed in ice-cold RIPA Buffer (Cell Signaling Technology) and the lysates were sonicated at 30% amplification for 5 s 3 times and centrifuged (12,500 × *g*, 10 mins). Protein quantification was determined using the BCA assay (Pierce). Equal protein concentrations were loaded into each well. The lysates were resolved on 7.5–12% SDS-PAGE gels and transferred onto nitrocellulose or PVDF membranes. Following blocking in TBS buffer containing 2% milk and 2% BSA, the membranes were immunoblotted with primary antibodies followed by appropriate secondary antibodies. Primary antibodies against phospho-AMPKα T172 (cat# 2535), AMPKα (cat# 5832), ACC1 (cat# 3676), phospho-ACC1 S79 (cat# 11818), hexokinase I (cat# 2024), hexokinase II (cat# 2867), PFKP (cat# 8164), PKM1/2 (cat# 3190), PKM2 (cat# 4053), LDHA (cat# 3582), PDHA (cat# 3205), GAPDH (cat# 5174), and β-actin (cat# 8457) were from Cell Signaling (Danvers, MA, USA). Glut1 (cat# PA1-46152) and Glut4 (sc-7938) were from Invitrogen (Carlsbad, CA, USA) and Santa Cruz (Dallas, TX, USA), respectively. The blotted membranes were developed with either SuperSignal™ West Pico PLUS Chemiluminescent Substrate or SuperSignal™ West Femto Maximum Sensitivity Substrate (ThermoFisher Scientific). Stripping of blots was achieved with stripping buffer (Cat# 21059, Thermo Scientific). Comparison of pACC1 was measured in Image J using a fixed box size and calculating the integrated density, which was then expressed as a ratio of pACC/tACC for each sample. Ratios were then normalized to the average of all the control samples and represented as a single data point for each sample.

### EdU proliferation assay

Proliferation by EdU incorporation was performed as previously described [10]. Briefly, cells were seeded at 60k cells/well on a 24-well plate, overnight followed by indicated treatments. For detection, culture media was spiked with 10 µM EdU (A10044 ThermoFisher) and mixed. After 6 h of incubation with EdU, cells were trypsinized into single cell suspension and fixed overnight at 4^o^C in 5% buffered formalin. The cells were then stained with propidium iodide, incubated with azide dye, copper sulfate, and ascorbic acid, and then analyzed for 530 nm and 695 nm emission by flow cytometry. Cells were gated for singlets, then propidium-iodide positivity, and percent positive for EdU was taken from the propidium-iodide-positive single cells.

### Fatty acid oxidation (FAO)

FAO activity was measured according to the manufacturer’s protocol (BRS, cat# E-141). Briefly, 200 K cells were seeded in a 12-well plate in quadruplicates and treated with DMSO, AdipoRon (25 µM), or Metformin (10 mM) for 6 h. Cell lysate was prepared using the lysis buffer provided in the kit. After BCA quantification, an equal amount of protein was loaded in a 96-well plate and incubated for 30 min with control or substrate solution, followed by measurement of optical density at 492 nm and activity calculation according to the manual.

### 2-NBDG (2-deoxy-2-[(7-nitro-2,1,3-benzoxadiazol-4-yl) amino]-D-glucose) uptake

For 2NBDG-uptake assay, cells were seeded in either a 96-well plate for spectrophotometric analysis or a 24-well plate for flow-cytometric analysis. The cells were treated with AdipoRon (25 µM), and then glucose- and serum-starved for 40 min prior to incubation with 2NBDG (200 µM, Cayman Chemical, Cat# 11046) in PBS for 80 min. At the endpoint, cells were washed with PBS and 2NBDG content was measured via plate reading at excitation 475 nm, emission 550 nm or flow analysis.

### Colony-formation assay

In total, 700 cells of the indicated cell lines were seeded in triplicate and allowed to attach to the plate. Then, the cells were treated as indicated for 36 h with indicated compounds. Surviving colonies were then maintained in drug-free media for 7 days; the media was refreshed every two days. At the endpoint, the colonies were stained with crystal violet solution (cat# V5265, Sigma) for 20 min. After imaging the plates, the colonies were dissolved in 10% acetic acid and OD read at 590 nm.

### Samples and statistics analysis

Samples sizes were chosen to achieve a minimum of triplicates for all experiments. Seahorse outlier wells were removed if ≥3 timepoints fell outside the range of 2*IQR +/− upper/lower quartile, or the results were biologically impossible (e.g., maximal respiration being significantly below baseline). For assessment of statistical significance, ordinary one-way ANOVA with Dunnett’s multiple-comparison test or Welch’s unpaired 2-way *t*-test was used when appropriate and indicated. All statistical tests were performed using GraphPad Prism5 software where *p < 0.05, **p < 0.01, ***p < 0.001, and ****p < 0.0001.

### Reporting summary

Further information on experimental design is available in the Nature Research Reporting Summary linked to this paper.

## Supplementary information

Supplementary files

Reporting Summary Checklist

## Data Availability

Figures for this paper were generated from raw data files that are available from the corresponding author on reasonable request.
