# Correction: Synthetic adiponectin-receptor agonist, AdipoRon, induces glycolytic dependence in pancreatic cancer cells

**DOI:** 10.1038/s41419-022-04615-0

**Published:** 2022-02-21

**Authors:** Sharon J. Manley, Appolinaire A. Olou, Jarrid L. Jack, Mariana T. Ruckert, R. McKinnon Walsh, Austin E. Eades, Bailey A. Bye, Joe Ambrose, Fanuel Messaggio, Shrikant Anant, Michael N. VanSaun

**Affiliations:** 1 Department of Cancer Biology, University of Kansas Medical Center, Kansas City, KS USA; 2 Department of Surgery, University of Miami Miller School of Medicine, Sylvester Comprehensive Cancer Center, Miami, FL USA

**Keywords:** Pancreatic cancer, Cell growth, Biologics, Pancreatic cancer

Correction to: *Cell Death and Disease* 10.1038/s41419-022-04572-8, published online 4 February 2022

The original version of this article unfortunately needed to be updated. A related file was included as supplementary and the author would like it removed. The original article has been corrected.

