# Supplementary material for: Synthetic adiponectin-receptor agonist, AdipoRon, induces glycolytic dependence in pancreatic cancer cells
Source: Cell Death Dis. 2022 Feb 4;13(2):114. doi: 10.1038/s41419-022-04572-8 (PMC8817044; doi:10.1038/s41419-022-04572-8)
Supplement: Supplementary file 1 — Supplementary files [file 41419_2022_4572_MOESM1_ESM.pdf]

Supplementary Figure 1, related to Figure 1: AdipoRon treatment induces extracellular acidification.

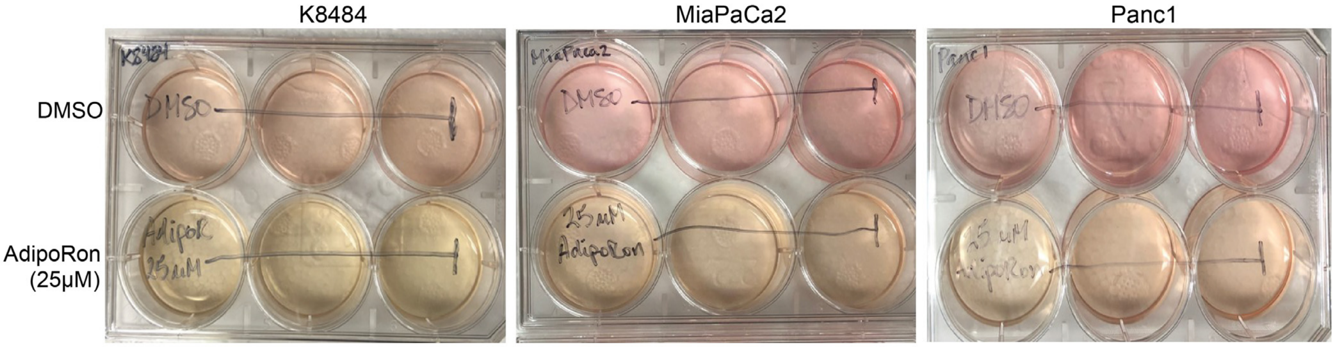

Treatment of pancreatic cancer cell lines with AdipoRon (25uM) leads to acidification (yellowing) of the media after 48 hours in comparison to control, DMSO-treated cells, n=3.

Supplementary Figure 2, related to Figure 4: Changes in glucose transporter types 1 and 4 upon AdipoRon (AR) treatment of PDAC cell lines

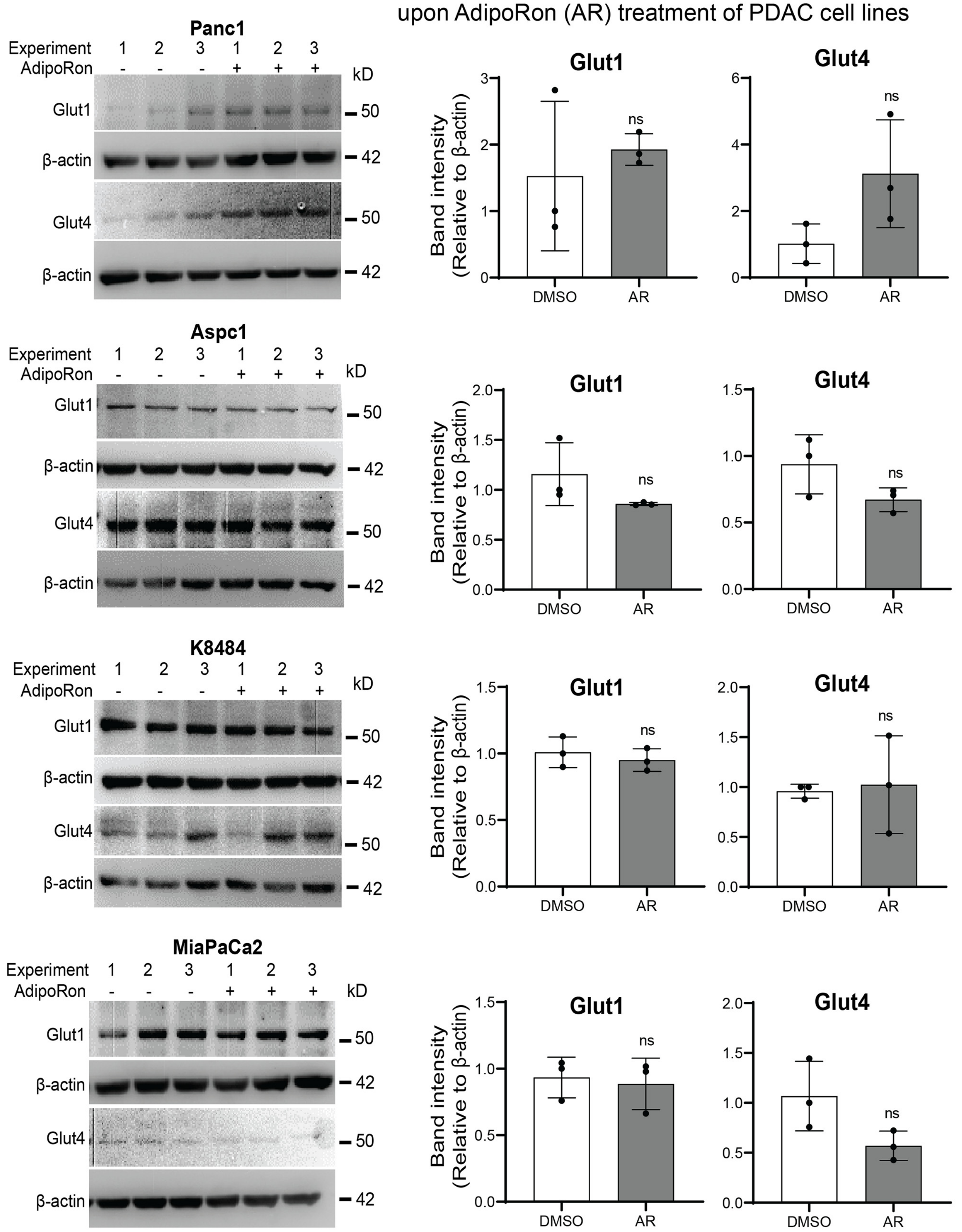

Treatment of pancreatic cancer cell lines with AdipoRon (25μM) for 24 hours led to variable cell line dependent changes in Glut1 or Glut4 levels. Band intensity for each receptor was compared to actin from the same sample and represented as a single data point. Students' t-test, N=3; * $p < 0.05$.

Supplementary Figure 3, related to Figure 4: AdipoRon treatment induces glucose uptake but not fatty acid oxidation.

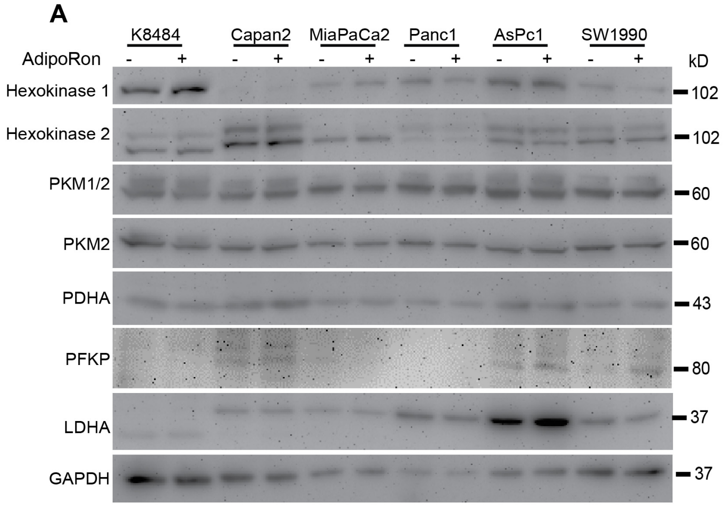

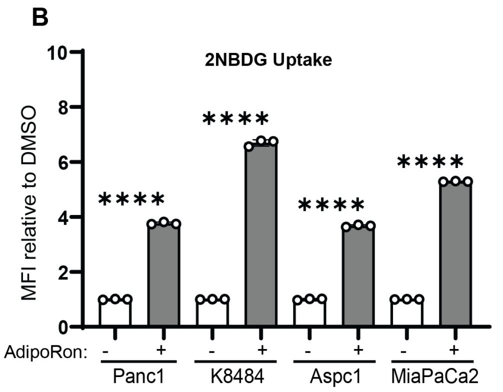

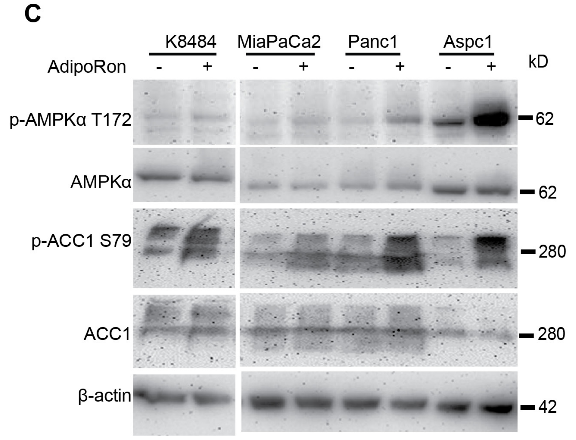

(A): Levels of molecular mediators of glucose metabolism were assessed after AdipoRon treatment (25μM). (B): Uptake of 2NBDG was assessed from flow histograms for PDAC cell lines after control (-) or AdipoRon (+) treatment. (C): Levels of p-AMPKα, tAMPKα, p-ACC1, and tACC1 in PDAC cell lines with or without AdipoRon treatment. (B, Students' t-test; n=3 * P < 0.05).

Supplementary Figure 4, related to Figure 5: AdipoRon enhances growth suppression in low glucose conditions

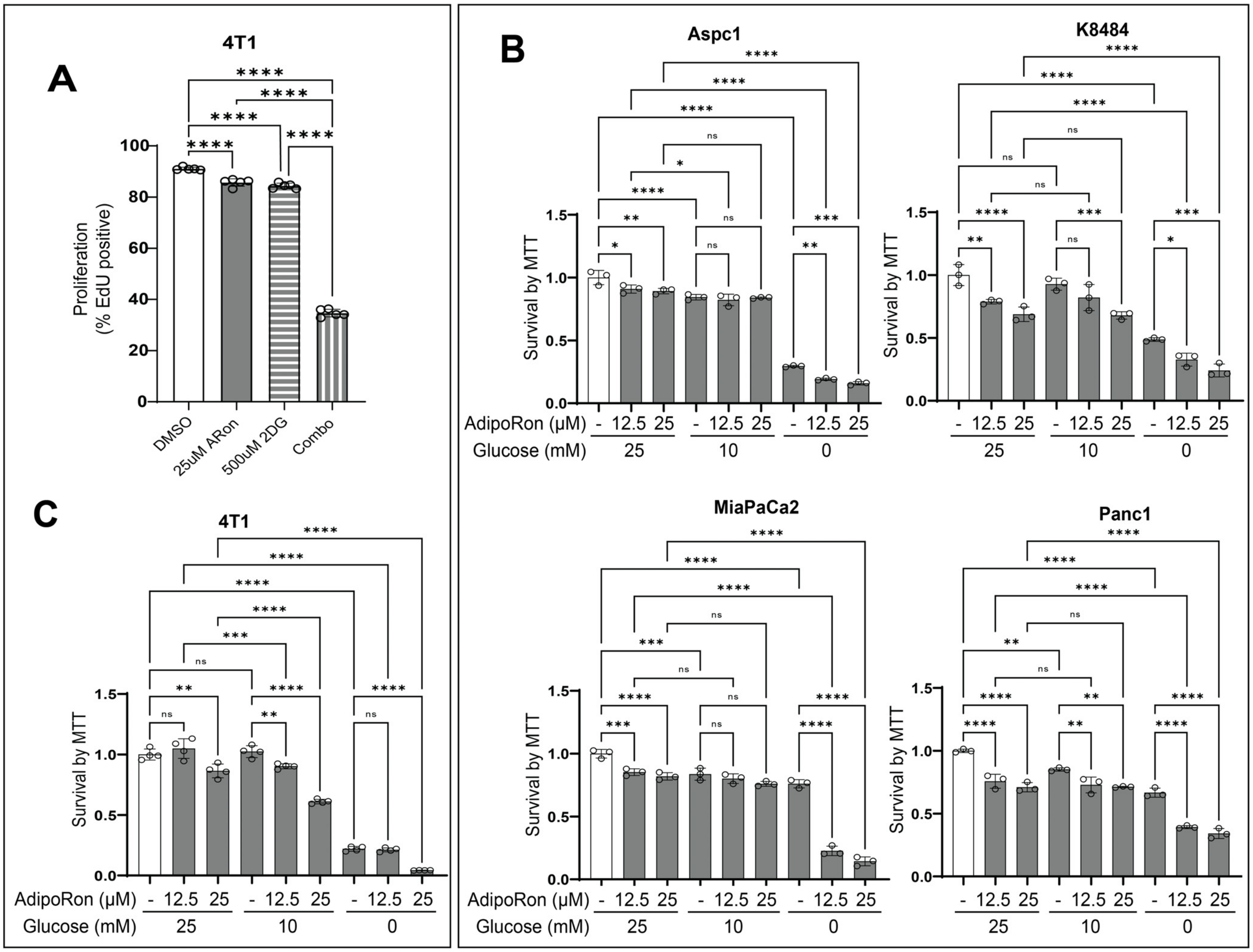

(A): 4T1 cells were treated alone or in combination with AdipoRon and/or the glycolysis inhibitor 2DG for 24 hours and then proliferation was measured by EdU incorporation; n=5.
(B & C): Indicated pancreatic cancer cell lines and breast cancer cell line(4T1) were treated with either DMSO (-) or AdipoRon (12.5 or 25µM) in standard (25mM), low glucose (10mM), or glucose-free (0mM) media alone or in combination for 48 hours followed by measurement of cell viability using the MTT assay. One-way ANOVA,* $P < 0.05$; (B, n=3; C, n=4)

Table: Similarities and Differences between the PDAC cell lines used in the study

PDAC Cell	K8484 (murine)	Capan2	MiaPaCa2	Panc-1	Aspc1	SW1990
Source	Primary	Primary	Primary	Primary	Metastatic	Metastatic
Mutation(s)	KRAS G12D, mut p53	KRAS G12V	KRAS G12C, mut p53, dCDK	KRAS G12D, mut p53, dCDK	KRAS G12D, mut p53, dCDK	KRAS G12D, wt p53, dCDK
